# Impact of *Bacillus subtilis* Antibiotic Bacilysin and *Campylobacter jejuni* Efflux Pumps on Pathogen Survival in Mixed Biofilms

**DOI:** 10.1128/spectrum.02156-22

**Published:** 2022-08-08

**Authors:** A. Erega, P. Stefanic, T. Danevčič, S. Smole Možina, I. Mandic Mulec

**Affiliations:** a Department of Food Science and Technology, Biotechnical Faculty, University of Ljubljana, Ljubljana, Slovenia; b Department of Microbiology, Biotechnical Faculty, University of Ljubljana, Ljubljana, Slovenia; c Chair of Microprocess Engineering and Technology/COMPETE, University of Ljubljana, Ljubljana, Slovenia; Ohio State University

**Keywords:** antibiotics, *Bacillus subtilis*, *Campylobacter jejuni*, bacillaene, bacilysin, biofilm formation, efflux pumps, secondary metabolites

## Abstract

The foodborne pathogen Campylobacter jejuni is typically found in an agricultural environment; in animals, such as birds, as an intestinal commensal; and also in food products, especially fresh poultry meat. Campylobacter interactions within mixed species biofilms are poorly understood, especially at the microscale. We have recently shown that the beneficial bacterium Bacillus subtilis reduces C. jejuni survival and biofilm formation in coculture by secreting the antibiotic bacillaene. We extend these studies here by providing evidence that besides bacillaene, the antagonistic effect of B. subtilis involves a nonribosomal peptide bacilysin and that the fully functional antagonism depends on the quorum-sensing transcriptional regulator ComA. Using confocal laser scanning microscopy, we also show that secreted antibiotics influence the distribution of C. jejuni and B. subtilis cells in the submerged biofilm and decrease the thickness of the pathogen’s biofilm. Furthermore, we demonstrate that genes encoding structural or regulatory proteins of the efflux apparatus system (*cmeF* and *cmeR*), respectively, contribute to the survival of C. jejuni during interaction with B. subtilis PS-216. In conclusion, this study demonstrates a strong potential of B. subtilis PS-216 to reduce C. jejuni biofilm growth, which supports the application of the PS-216 strain to pathogen biofilm control.

**IMPORTANCE** Campylobacter jejuni is a prevalent cause of foodborne infections worldwide, while Bacillus subtilis as a potential probiotic represents an alternative strategy to control this alimentary infection. However, only limited literature exists on the specific mechanisms that shape interactions between B. subtilis and C. jejuni in biofilms. This study shows that in the two species biofilms, B. subtilis produces two antibiotics, bacillaene and bacilysin, that inhibit C. jejuni growth. In addition, we provide the first evidence that specific pathogen efflux pumps contribute to the defense against B. subtilis attack. Specifically, the CmeDEF pump acts during the defense against bacilysin, while CmeR-dependent overexpression of CmeABC nullifies the bacillaene attack. The role of specific B. subtilis antibiotics and these polyspecific pumps, known for providing resistance against medically relevant antibiotics, has not been studied during bacterial competition in biofilms before. Hence, this work broadens our understanding of mechanisms that shape antagonisms and defense during probiotic-pathogen interactions.

## INTRODUCTION

The Gram-negative, foodborne pathogen Campylobacter jejuni is typically found in animals, such as broiler chickens, where it is an intestinal commensal, and also in food products, especially fresh poultry meat and contaminated drinking water (1, 2). C. jejuni is the most common cause of human campylobacteriosis and a consistent and worsening food safety problem (zoonosis) in developed European Union countries and globally (3–6). Persistent Campylobacter communities in agricultural, industrial poultry, and husbandry surfaces/environments facilitate their circulation in the broiler gastrointestinal tract (GIT) (7), resulting in contaminated food products (2, 8). Hence, novel strategies, particularly in the agricultural, poultry, and food industries (9–13), are needed and an active effort in developing probiotics to reduce Campylobacter colonization in poultry is also required (14–17).

Bacillus subtilis has been applied commercially as a probiotic (18–20) to control foodborne pathogens and with a beneficial effect on the GIT microbial balance and gut health of broilers (19, 21, 22). B. subtilis is also a model organism used for biofilm research (15, 23–25) and is known for producing a plethora of secondary metabolites (26, 27). It has been demonstrated that it carries the potential to prevent or destroy enteric bacterial growth, biofilms, or adhesion to inert surfaces (15, 28–33). However, many questions about mechanisms shaping interactions of B. subtilis with foodborne pathogens remain unanswered, especially at the microscale. We have recently provided evidence of strong antibiofilm activity of the B. subtilis PS-216 strain against C. jejuni (15) and confirmed its antagonism against C. jejuni in sterile chicken intestinal content (34). The strongest inhibition was achieved under conditions representing a chicken environment (42°C, microaerobic atmosphere, and chicken litter medium), and even if C. jejuni initial counts surpassed B. subtilis PS-216 by 1,000-fold, this strain still inhibited the growth of the pathogen (34). These results support the use of B. subtilis PS-216 as a promising biocontrol strain and warrant further studies addressing the mechanisms and consequences of C. jejuni-B. subtilis interactions.

Our previous study developed the *in vitro* model of C. jejuni and B. subtilis interaction in a biofilm setting (15). In brief, we investigated the spatial distribution of the probiotic and pathogen during biofilm formation at the microscale using confocal laser scanning microscopy (CLSM) and fluorescently labeled strains, and we showed that the production of bacillaene significantly affected pathogen biofilm formation (15). However, abolishing bacillaene production did not completely abolish the antagonistic potential of B. subtilis (15), suggesting that other secreted factors may play a role. We hypothesized that the response regulator protein (ComA) of the ComQXPA quorum-sensing system (35, 36) is involved in the antagonism of B. subtilis. ComA positively affects the expression of the *pks* gene cluster relevant for the synthesis of the polyketide antibiotic bacillaene (37): the *bac* operon involved in nonribosomally synthesized dipeptide antibiotic bacilysin (38) and the *srfA* operon responsible for the surfactin synthesis in B. subtilis monocultures (39). However, to our knowledge, whether ComA and the three ComA-dependent antibiotics affect C. jejuni growth and biofilm formation has not been resolved. We tested this using static biofilm assays, which provide useful means to study biofilms (40–42), allowing analyses by confocal microscopy (43) and conditions suitable for the growth of Campylobacter biofilms (41, 42) at 42°C under microaerobic conditions, which represent the normal physiological state of the broilers most commonly infected by C. jejuni (44).

Bacterial multidrug efflux pumps constitute an important class of resistance determinants against various medically important antibiotics (45, 46); hence, they also contribute to the antibiotic resistance of C. jejuni (47). This pathogen can mobilize three efflux systems to fight an antibiotic attack (48). (i) The main CmeABC efflux pump, belonging to the resistance nodulation (RND) family (48–51), contributes to the resistance of a broad range of antibiotics (52) and consists of an inner membrane transporter protein (CmeB), a periplasmic membrane fusion protein (CmeA), and an outer membrane factor (CmeC). Mutations in this tripartite system effect drug susceptibility (46, 49). (ii) The second RND efflux system, CmeDEF, which plays a supporting role to CmeABC and has been less studied (53), involves CmeD as an outer membrane channel protein, CmeE as a periplasmic fusion protein, and CmeF as an inner membrane transporter (48). (iii) The major facilitator superfamily (MFS), CmeGH, is involved in the resistance to antibiotics such as erythromycin, tetracycline, gentamicin, and others (54). Expression of both RND efflux pumps has been observed in clinical C. jejuni isolates from humans and poultry are resistant to antibiotics (55). Although efflux pumps are important for antibiotic resistance (49, 51, 56) and even biofilm formation in different bacterial species (57, 58), only a few studies have addressed their role in bacterial interactions during coincubation with other microorganisms (59–61), and there is only one study addressing interactions between C. jejuni and Acanthamoeba polyphaga (61). To our knowledge, the role of C. jejuni efflux pumps has not yet been investigated in cocultures with antagonistic bacteria, such as B. subtilis or any other potential probiotic bacteria.

This study investigates the effects of ComA-dependent secreted antibiotics of B. subtilis, namely, bacillaene, surfactin, and bacilysin, on C. jejuni growth by using a static biofilm assay. In addition, it addresses the role of the C. jejuni efflux systems CmeABC, CmeDEF, and CmeGH and the transcriptional repressor CmeR in the survival of C. jejuni during interaction with B. subtilis PS-216. Altogether, we provide evidence that the antagonism of B. subtilis PS-216 against C. jejuni depends on ComA and two secreted antibiotics controlled by ComA. We also show that C. jejuni RND efflux systems contribute to the survival of this pathogen in coculture with B. subtilis PS-216. Moreover, the results suggest that the CmeDEF efflux pump contributes to the defense against bacilysin and the CmeR regulator against bacillaene.

## RESULTS

### The *B. subtilis* antibiotics bacillaene and bacilysin mediate anti-*Campylobacter* activity.

Our previous work highlighted the critical role of 4′-phosphopantetheinyl transferase (*sfp*) and polyketide (bacillaene) synthesis (*pks*) genes in the effect of B. subtilis on C. jejuni that resulted in disrupted growth and biofilm formation during coculture biofilm assay (15). However, the anti-Campylobacter effect of B. subtilis PS-216 was not completely abolished in the *pks* mutant, suggesting that the PS-216 effect is due to the production of at least two antimicrobial compounds.

**FIG 1:**
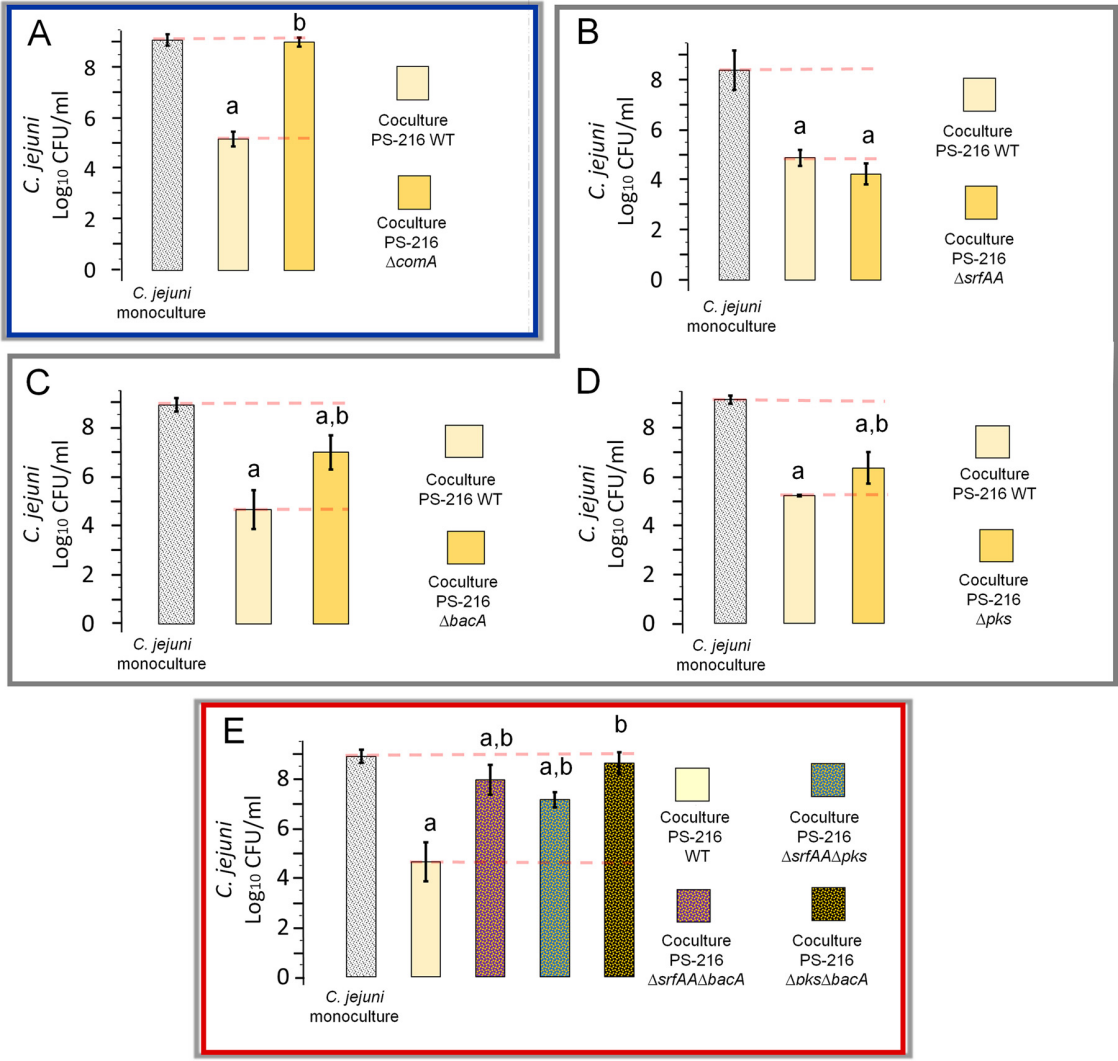
Inhibition of C. jejuni growth by B. subtilis PS-216 mutants in loci involved in the synthesis of the antibiotics bacillaene and bacilysin. PS-216 mutations are in loci involved in nonribosomal/polyketide synthesis (bacillaene [*pks*], bacilysin [*bacA*], and surfactin [*srfAA*]) and transcriptional regulatory protein ComA. (A) C. jejuni during mono- and coculture with B. subtilis mutant in the *comA* gene encoding transcriptional regulatory protein ComA. (B) C. jejuni during mono- and coculture with B. subtilis mutant in *srfAA* gene involved in nonribosomal peptide synthesis of surfactin. (C) C. jejuni during mono- and coculture with B. subtilis mutant in *bacA* gene in nonribosomal peptide synthesis of bacilysin. (D) C. jejuni during mono- and coculture with B. subtilis mutant in *pks* locus involved in polyketide synthesis of bacillaene. (E) C. jejuni during mono- and coculture with B. subtilis double mutants in loci involved in polyketide synthesis of bacillaene as nonribosomal synthesis of surfactin and bacilysin. All cocultures were grown in MHB medium under static microaerophilic conditions at 42°C for 24 h. Samples containing biofilm and broth were vortexed prior to plating. The results are presented as colony counts. Three biological and up to three technical repeats were used. The error bars represent the standard deviation of the mean. “a” and “b” represent statistically significant values, where “a” represents hypothesis testing between C. jejuni monoculture and C. jejuni in coculture with B. subtilis (mutant strains and WT), and “b” represents hypothesis testing between C. jejuni in coculture with B. subtilis mutant and C. jejuni in coculture with B. subtilis WT. Data were statistically evaluated using a two-sample *t* test (see Materials and Methods for details).

To find candidate genes responsible for the antimicrobial effect of B. subtilis toward C. jejuni observed in our previous work, we focused on a regulatory gene (*comA*) and genes involved in secondary metabolism (*pks*, *bacA*, and *srfAA*). We hypothesized that strains carrying mutations in *comA*, *pks* (bacillaene), *bacA* (bacilysin), and *srfAA* (surfactin) would exert a diminished inhibitory effect against C. jejuni in coculture assays compared to the wild-type strain (PS-216 WT). First, we generated mutations by inserting an antibiotic resistance cassette into each of these genes; second, we generated double mutations in surfactin-bacillaene (Δ*srfAA* Δ*pks*), surfactin-bacilysin (Δ*srfAA* Δ*bacA*), and bacillaene-bacilysin (Δ*pks* Δ*bacA*) (Table 1). The inhibitory effect of each B. subtilis mutant strain in coculture with C. jejuni NCTC 11168 at the ratio of 1:10 was measured as the colony counts after 24 h of coincubation. In comparison to B. subtilis PS-216 WT, the Δ*comA* mutant showed no inhibition of C. jejuni (Δ*comA*, *p* = 3.53 × 10^−7^) as C. jejuni CFU counts in coculture with the Δ*comA* mutant were comparable to C. jejuni counts in monoculture (*p* = 0.58) (Fig. 1A). In contrast, the inhibition of B. subtilis Δ*srfAA* mutant was similar to the inhibition of PS-216 WT and both strains inhibited the growth of C. jejuni significantly (*p* = 5.40 × 10^−4^) (Fig. 1B). These results imply that ComA, but not surfactin, which is ComA regulated, mediates C. jejuni inhibition. In contrast to the Δ*srfAA* mutant, a strain carrying a mutation in two other ComA-regulated genes (Δ*bacA* and Δ*pks*) showed significantly lower inhibition of C. jejuni (Δ*bacA*, *p* = 3.56 × 10^−4;^ Δ*pks*, *p* = 0.0076) than PS-216 WT (*p* = 0.0024) (Fig. 1C and D). Moreover, the Δ*srfAA* Δ*bacA* and Δ*srfAA* Δ*pks* double mutants also showed significantly lower inhibition of C. jejuni compared to the PS-216 WT, with an inhibition of 0.95 log_10_ CFU/mL (*p* = 2.30 × 10^−5^) and 1.75 log_10_ CFU/mL (*p* = 5.0 × 10^−5^), respectively. It is important to note that both double mutants still reduced the CFU counts of C. jejuni significantly compared to C. jejuni monoculture CFU counts (Δ*srfAA* Δ*bacA*, *p* = 0.035), (Δ*srfAA* Δ*pks*, *p* = 8.6 × 10^−6^) (Fig. 1E). The lowest C. jejuni inhibition (compared to the PS-216 WT) was observed when C. jejuni was cocultured with the double mutant Δ*pks* Δ*bacA* (inhibition of 0.27 log_10_ CFU/mL, *p* = 7.6 × 10^−7^). The CFU count of C. jejuni in coculture with the Δ*pks* Δ*bacA* double mutant was similar to the CFU count of C. jejuni in monoculture (*p* = 0.25) (Fig. 1E), implying that the major antibacterial effect of B. subtilis PS-216 lies within these two loci.

**TABLE 1:** Strains used in this study

Strain or plasmid	Strain abbreviation	Background	Genome description	Source or reference(s)
Strains				
C. jejuni subsp*. jejuni*				
NCTC11168	WT		Domesticated strain	78, 79
NCTC11168	WT-GFP	NCTC11168	pWM1007	76
NCTC11168		NCTC11168	Δ*cmeB*::*kn* (Kn)	75
NCTC11168		NCTC11168	Δ*cmeF*::*cm* (Cm)	75
NCTC11168		NCTC11168	Δ*cmeR*::*cm* (Cm)	75
NCTC11168		NCTC11168	Δ*cmeG*::*kn* (Kn)	74
B. subtilis				
PS-216	WT		Undomesticated strain	77
BM1707		PS-216	Δ*srfAA*	15
BM1875		PS-216	Δ*pks*::*spec* (Spec)	This study
BM1887		PS-216	Δ*bacA*::*erm* (Erm)	This study
BM1403		PS-216	Δ*comA*::*erm* (Erm)	This study
BM1888		PS-216	Δ*srfAA* Δ*bacA*::*erm* (Erm)	This study
BM1889		PS-216	Δ*srfAA* Δ*pk*s::*spec* (Spec)	This study
BM1890		PS-216	Δ*pks*::*spec* Δ*bacA*::*erm* (Erm, Spec)	This study
BM1629	WT-RFP	PS-216	*sacA*::P_43_*-mkate2* (Kn)	72
BM1894	Δ*pks*-RFP	PS-216	Δ*pks*::*spec sacA*::P_43_*-mkate2* (Kn)	This study
BM1903	Δ*bacA-*RFP	PS-216	Δ*bacA*::*erm sacA*::P_43_*-mkate2* (Kn)	This study
BM1896	Δ*pks *Δ*bacA-*RFP	PS-216	Δ*pks*::*spec* Δ*bacA*::*erm sacA*::P_43_*-mkate2* (Kn)	This study
DNA donors for transformation				
BKE37740		168 *trpC2*	Δ*bacA*::*erm* (Erm)	70
BD1605		168	Δ*comA*::*erm* (Erm)	73
PSK0178		3610	Δ*pks*::*spec* (Spec)	71
Plasmid (from *E. coli* strains)				
pMS17		EM1096	*sacA*::P_43_*-mkate2* (Kn)	72

Moreover, in coculture with C. jejuni the growth of the B. subtilis Δ*comA* (*p* = 0.43), the Δ*srfAA* mutant (*p* = 0.32), the Δ*pks* mutant (*p* = 0.23), or the PS-216 Δ*pks* Δ*bacA* double mutant (*p* = 0.088) was not affected (see Fig. S1A, B, and D in the supplemental material). In contrast, when cocultured with C. jejuni the growth of the *bacA* mutant was reduced (*p* = 0.0038), as was the growth of the Δ*srfAA* Δ*bacA* and Δ*srfAA* Δ*pks* double mutants with inhibitions of 0.52 log_10_ CFU/mL (*p* = 0.011) and 0.60 log_10_ CFU/mL (*p* = 0.0034), respectively (see Fig. S1C and E).

### The *B. subtilis* antibiotics bacillaene and bacilysin prevented *C. jejuni* biofilm formation.

**FIG 2:**
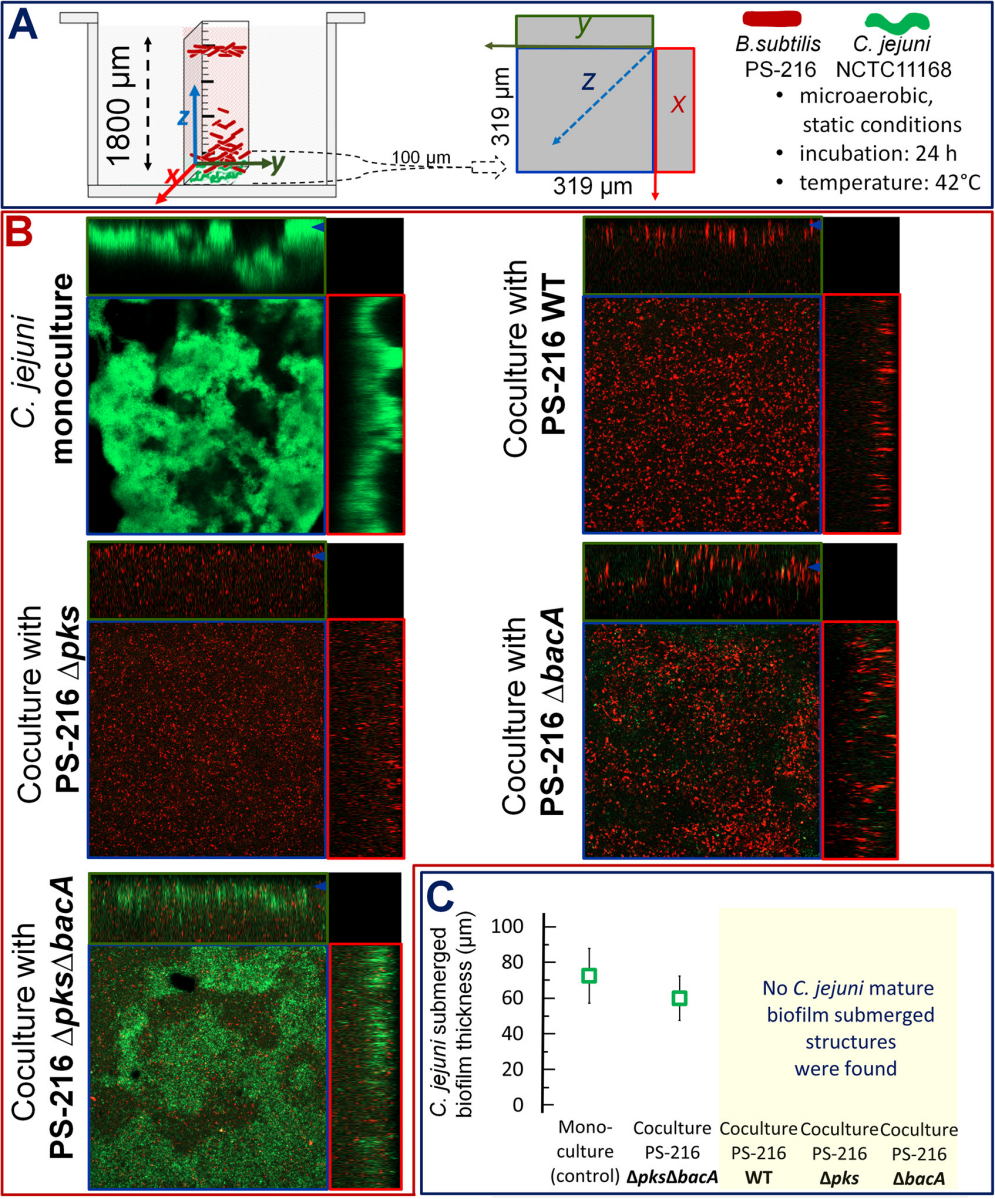
B. subtilis antibiotics bacillaene and bacilysin are antibiofilm mediators preventing C. jejuni from forming a submerged biofilm. (A) Schematic representations of an experimental model for investigating interactions between a pathogen (C. jejuni) and a potential antagonist (B. subtilis) under static conditions at 42°C in MHB medium using CLSM in the total volume of the well (left, height 1,800 μm of the well; right, schematic representing the ortho visualization of the submerged biofilm). The ortho view depicts the fluorescence in each cut section related to the *x*, *y*, and *z* dimensions of the submerged biofilm. The colored boxes (blue, red, green) each represent a different view through the biofilm. The larger panel labeled “*z*” is a two-dimensional distribution of the submerged biofilm in *x-y* dimension, where only the bottom *z* stack (3.5 μm) is presented. While the smaller side panels (*x* and *y*) represent combined *z* stacks through 100-μm depth of the submerged biofilm. (B) The CLSM images represent C. jejuni submerged biofilms incubated for 24 h in static microaerobic conditions at 42°C as a monoculture (control) compared to the phenomenon observed in coculture with PS-216 WT, PS-216 Δ*pks*, PS-216 Δ*bacA*, and PS-216 Δ*pks* Δ*bacA* strains. (C) Effect of 24 h of cultivation time on C. jejuni submerged biofilm formation expressed as biofilm thickness (μm). The results show the means and standard deviations for five independent experiments. Data were statistically evaluated using the Mann-Whitney test (see Materials and Methods for details). For CLSM analysis, we performed five biological experiments with five technical replicates (five wells). CLSM analysis was performed in three different position spots in each well where biofilms were grown.

The experiment described above shows that the most potent antibacterial effect of B. subtilis PS-216 against C. jejuni depends on *pks* and *bac* loci. In order to further investigate the effect of the B. subtilis PS-216 antibiotics bacillaene (*pks*) and bacilysin (*bacA*) on submerged C. jejuni biofilm thickness, single (Δ*bacA* or Δ*pks*) and double (Δ*bacA* Δ*pks*) B. subtilis knockout mutants were cocultured with C. jejuni NCTC 11168 at 42°C under microaerobic and static conditions, and the effects were compared to those of the PS-216 WT (Table 1 and Fig. 2A). First, C. jejuni monoculture (control) formed a submerged biofilm, where cells were gathered in aggregates and were partially attached to the bottom of the well, forming characteristic submerged biofilm finger-like structures (Fig. 2B, top left). Second and as expected, the presence of B. subtilis PS-216 WT showed a strong inhibitory effect on C. jejuni submerged biofilm formation. We did not detect any visible submerged biofilm structures or cell aggregates of C. jejuni (green dots) (Fig. 2B, top right). Similarly, the same inhibitory effect on biofilm formation was observed when C. jejuni was cocultured with the B. subtilis PS-216 Δ*pks* mutant (Fig. 2B, middle left). However, in coculture with the PS-216 Δ*bacA* mutant C. jejuni formed small cell aggregates (groups of green dots) (Fig. 2B, middle right). In line with these CFU experiments, coculture of C. jejuni with Δ*pks* Δ*bacA* PS-216 had no inhibitory effect on C. jejuni biofilm formation, and submerged biofilm finger-like structures were preserved (Fig. 2, bottom left).

In addition, the thickness of submerged biofilms was analyzed by three-dimensional (3D) confocal microscopy imaging, which confirmed a similar biofilm thickness of C. jejuni in coculture with the B. subtilis Δ*pks* Δ*bacA* mutant (59.90 μm ± 12.44 μm) and in C. jejuni monoculture (72.50 μm ± 15.30 μm) (*p* = 4.7 × 10^−5^, nonparametric test). The biofilm thickness of C. jejuni in coculture with PS-216 WT, PS-216 Δ*pks*, and PS-216 Δ*bacA* strains was not possible to quantify by this approach due to too-strong growth inhibition (Fig. 2C). Although we could still detect green clusters of C. jejuni in the coculture with the PS-216 Δ*bacA* mutant, which were not visible in the coculture with the PS-216 WT strain or the PS-216 Δ*pks* mutant, these cell clusters were very sporadic and did not form a homogenous biofilm. Based on differences in C. jejuni biofilm thickness and on confocal images of its submerged biofilm in coculture with B. subtilis WT and the mutants, we concluded that bacilysin has a stronger inhibitory effect on biofilm formation than bacillaene.

In contrast, all of the B. subtilis strains tested formed visible submerged biofilms at the bottoms of the wells in mono- and cocultures with C. jejuni NCTC 11168 (Fig. 2B; see also Fig. S2). Although B. subtilis cell clusters were visible in all cocultures, we observed some morphological differences. For example, B. subtilis clusters were less prominent in PS-216 Δ*pks* Δ*bacA* submerged biofilm during coculture with C. jejuni NCTC 11168 (Fig. 2B; see also Fig. S2), suggests that the production of antibiotics may promote the fitness of the producer in a mixed biofilm with C. jejuni. Moreover, we observed that mutations in antibiotic-producing loci contribute to the PS-216 biofilm phenotype even in monocultures, with the Δ*pks* and Δ*pks* Δ*bacA* mutants forming morphologically different and less-prominent submerged biofilms if grown alone (see Fig. S2). This observation is consistent with recently the results of Li et al. (62), who show that bacillaene may enhance the biofilm formation of *Bacillus* spp.

### Efflux apparatus systems of *C. jejuni* improve survival during interaction with PS-216 in coculture.

**FIG 3:**
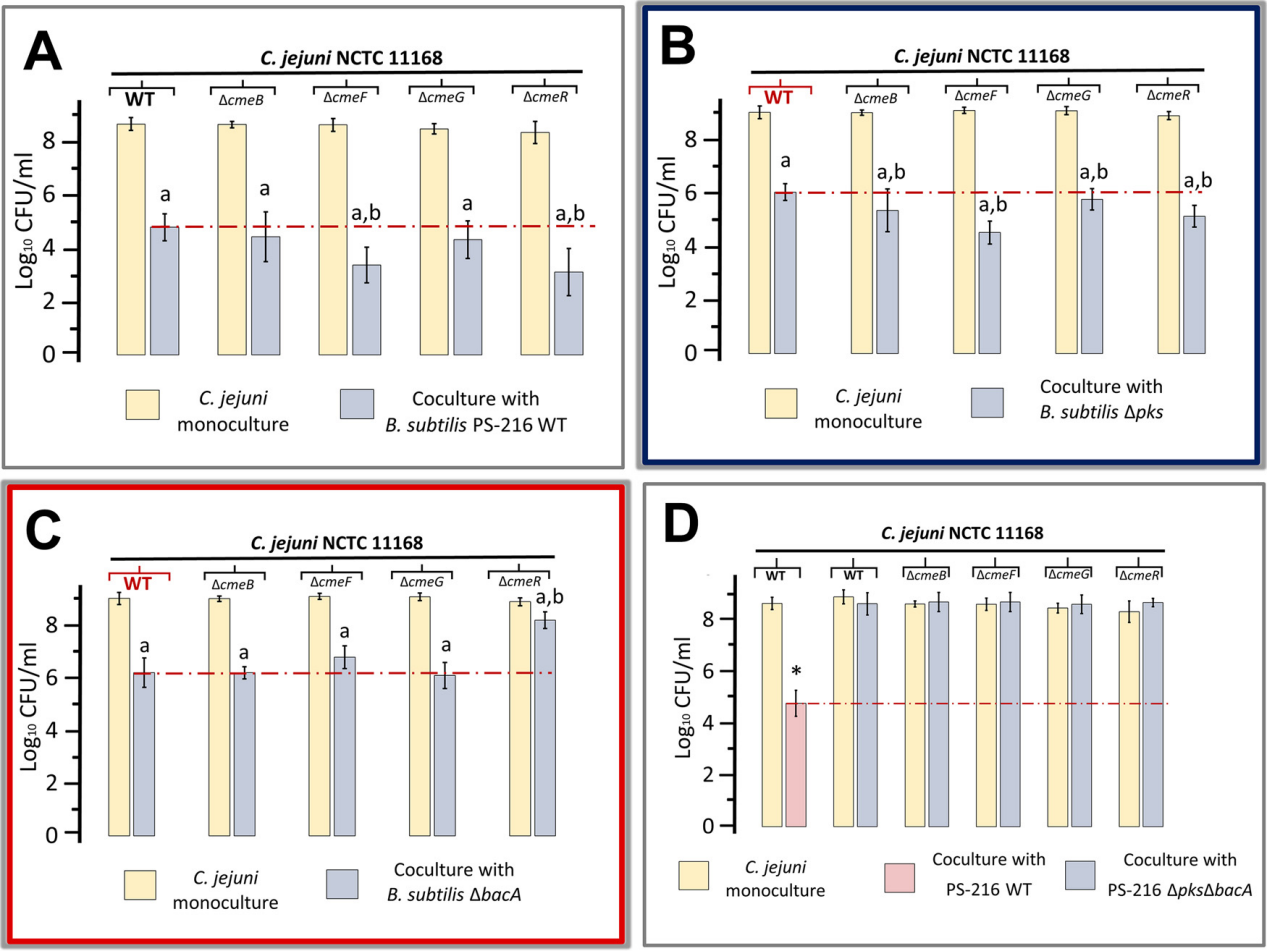
C. jejuni loci for efflux apparatus system contribute to the defense against *B. subtilis* PS-216 in coculture. The growth of C. jejuni WT and C. jejuni Δ*cmeB*, Δ*cmeF*, Δ*cmeG*, *ΔcmeR* efflux pump mutants was measured as colony counts after 24 h of incubation under static conditions at 42°C in MHB medium in monoculture (results in yellow columns) and coculture (results in gray columns) with the B. subtilis PS-216 WT strain (A), the PS-216 mutant lacking the locus for polyketide antibiotic bacillaene (PS-216 Δ*pks*) (B), the PS-216 mutant not producing the dipeptide antibiotic bacilysin (PS-216 Δ*bacA*) (C), and the PS-216 mutant lacking loci for both antibiotics: bacillaene and bacilysin (PS-216 Δ*pks*Δ*bacA*) (D). In panel D, the PS-216 WT effect on C. jejuni growth (red column) was included. Experiments were performed in at least three (D), five (B and C), or eight (A) biological replicates. Each biological replicate was always performed in three technical replicates. Samples containing biofilm and broth were vortexed prior to plating. The results are presented as colony counts. The error bars represent the standard deviations of the mean. In panels B and C, “a” and “b” represents statistically significant values, where “a” represents hypothesis testing between C. jejuni monoculture and C. jejuni in coculture with B. subtilis, and “b” represents hypothesis testing between C. jejuni mutant in coculture with B. subtilis and C. jejuni WT in coculture with B. subtilis. In panel D, the asterisk (*) represents statistically significant values. Data were statistically evaluated using a two-sample *t* test (see Materials and Methods for details).

Both identified antagonists of C. jejuni presumably target intracellular processes. Bacillaene inhibits bacterial protein synthesis (63). Bacilysin induces the lysis of the microbial cell wall by inhibiting the intracellular enzyme glucosamine-6-phosphate synthase, and mannoprotein or peptidoglycan biosynthesis in fungi and bacteria, respectively (64). Pathogens, including C. jejuni, apply defense systems against an antibiotic attack that include different efflux pumps (46, 48, 49), but it we lack evidence how efflux pumps contribute to C. jejuni growth in mixed-species biofilms. Therefore, we tested the effects of Campylobacter efflux pumps (CmeABC, CmeDEF, and CmeGH) and the repressor CmeR on the pathogen’s survival in coculture with B. subtilis PS-216. Specifically, we tested four C. jejuni mutants: two mutants that lack the respective RND membrane transporter (Δ*cmeB* or Δ*cmeF*), the Δ*cmeG* mutant lacking the MFS efflux transporter, and the Δ*cmeR* mutant, which overproduces the CmeABC efflux pump (48, 51, 53, 54). These mutants were incubated in coculture with B. subtilis PS-216 at a 10:1 ratio and grown at 42°C in Müller-Hinton broth (MHB) medium under microaerobic conditions. Colony counts of both species were determined after 24 h of incubation. As expected, all four C. jejuni mutants lacking efflux pump genes (Δ*cmeB*, Δ*cmeF*, and Δ*cmeG*) and the repressor (Δ*cmeR*) were significantly inhibited by B. subtilis PS-216 WT compared to the growth of C. jejuni mutants in monoculture (*p* ≤ 0.05) (Fig. 3A). The inhibition of Δ*cmeF* (*p* = 2.86 × 10^−4^) and Δ*cmeR* (*p* = 5.60 × 10^−4^) with 5.24 log_10_ inhibition (Δ*cmeF*) and 5.23 log_10_ inhibition (Δ*cmeR*) was stronger than that of C. jejuni WT, which was ~4.0 log_10_ (Fig. 3A). In contrast, inhibition of the *ΔcmeB* and *ΔcmeG* mutants was not significantly different from that of the WT C. jejuni (Fig. 3A). None of the four tested C. jejuni efflux pump mutants affected the growth of B. subtilis PS-216 WT (*p*_24_ ≥ 0.05) (see Fig. S3A). This suggested that the CmeF but not CmeB membrane transporter positively contributed to the defense against the PS-216-produced antibiotics bacillaene and bacilysin.

Next, we tested the role of efflux pumps in the C. jejuni resistance against specific B. subtilis antibiotics. In order to do that, we first set up an experiment where each of the four C. jejuni efflux mutant strains (Δ*cmeB*, Δ*cmeF*, Δ*cmeG*, and Δ*cmeR*, respectively) were cocultured with the B. subtilis Δ*pks* mutant (lacking bacillaene) at a 10:1 ratio. The colony counts of both species were determined after 24 h of coincubation (Fig. 3B). The B. subtilis PS-216 Δ*pks* mutant strongly inhibited all four C. jejuni efflux mutant strains compared to the growth of C. jejuni mutants in monoculture (*p* ≥ 0.05) (Fig. 3B). The inhibitions of Δ*cmeF* and Δ*cmeR* mutants was significantly stronger (Δ*cmeF*, *p* = 4.85 × 10^−9^; Δ*cmeR p* = 1.20 × 10^−4^), with 4.60 log_10_ inhibition (Δ*cmeF*) and 3.80 log_10_ inhibition (Δ*cmeR*), than that of C. jejuni NCTC 11168 strain WT, with 2.98 log_10_ inhibition (Fig. 3B). The other two efflux pump mutant strains (Δ*cmeB* and Δ*cmeG*) were still inhibited by the B. subtilis PS-216 Δ*pks* mutant (Δ*cmeB*, *p* = 0.048; Δ*cmeG*, *p* = 0.037), but the effect was not significantly different from that of PS-216 WT (Fig. 3A). None of the four tested C. jejuni efflux pump mutants affected the growth of B. subtilis PS-216 Δ*pks* (*p* ≥ 0.05) (see Fig. S3B). These results are consistent with the conclusion presented above and point to the importance of CmeF in the defense against bacilysin and the negative role of CmeABC (overexpressed) in this defense.

Next, we cocultured C. jejuni efflux mutant strains with a B. subtilis Δ*bacA* strain lacking bacilysin. The B. subtilis Δ*bacA* mutant also inhibited all four C. jejuni efflux mutant strains compared to their monocultures (*p* ≤ 0.05) (Fig. 3C). Interestingly, the Δ*cmeR* mutant was less sensitive to inhibition by the PS-216 Δ*bacA* strain, with only a small drop of 0.70 log_10_ (*p* = 5.97 × 10^−7^), while the C. jejuni NCTC 11168 WT strain growth decreased by 2.82 log_10_ (Fig. 3C). A similar tendency, albeit much less striking, was visible with the C. jejuni efflux pump Δ*cmeF* mutant (2.30 log_10_ inhibition), but the effect was not significant (Δ*cmeF*, *p* = 0.051) (Fig. 3C). The other two efflux pump mutant strains (Δ*cmeB* and Δ*cmeG*) were inhibited by B. subtilis PS-216 Δ*bacA* to a similar extent as C. jejuni NCTC 11168 WT (Δ*cmeB*, *p* = 0.51; Δ*cmeG*, *p* = 0.28) (Fig. 3C). None of the four tested C. jejuni efflux pump mutants affected the growth of the B. subtilis PS-216 Δ*bacA* mutant (*p*_24_ ≥ 0.05) (see Fig. S3C). Overall, the lack of CmeR gave C. jejuni a significant advantage in competition against the PS-216 Δ*bacA* strain, suggesting that overexpression of CmeABC efflux pump provides resistance to bacillaene. In contrast, the lack of CmeR made C. jejuni more sensitive to bacilysin.

Finally, we set up an experiment where each C. jejuni efflux pump mutant strain was cocultured with the B. subtilis Δ*pks* Δ*bacA* mutant (lacking bacillaene and bacilysin) at a 10:1 ratio, and the colony counts of both species were determined after 24 h of coincubation under standard conditions (Fig. 3D). The Δ*pks* Δ*bacA* strain failed to inhibit all four efflux pump mutants (Δ*cmeB*, *p* = 0.99; Δ*cmeR*, *p* = 0.42; Δ*cmeF*, *p* = 0.60; and Δ*cmeG*, *p* = 0.32) (Fig. 3D), confirming their role in the C. jejuni defense against bacillaene and bacilysin. No significant influence on the growth of the B. subtilis Δ*pks* Δ*bacA* mutant was detected in coculture with the C. jejuni Δ*cmeB* (*p* = 0.21), Δ*cmeF* (*p* = 0.11), Δ*cmeG* (*p* = 0.17), and Δ*cmeR* (*p* = 0.23) mutants (see Fig. S3D).

## DISCUSSION

C. jejuni is one of the most common foodborne bacterial pathogens in humans and represents a consistent food safety problem in developed countries globally (1, 4). Survival of this pathogen is enhanced under stress and in biofilms (65), which emphasizes a need for active efforts to develop probiotics capable of reducing Campylobacter colonization in poultry to improve animal health (14, 16). This need also calls for a better understanding of molecular determinants driving pathogen-probiotic interactions.

Here, we extend our results on the control of C. jejuni biofilms by B. subtilis PS-216 (15) and the reported probiotic potential of PS-216 against C. jejuni in sterile chicken intestinal content (34) and in broilers (14). Specifically, we show here that two diffusible antibiotics the polyketide bacillaene and the dipeptide bacilysin, contribute to the antimicrobial/antibiofilm effects of PS-216 against C. jejuni in a static *in vitro* biofilm culture system. We report on the role of the transcriptional regulator ComA (35, 36) in the PS-216-driven antagonism and of C. jejuni RND efflux systems in the defense against it.

In B. subtilis, ComA controls the production of bacillaene (37), bacilysin (*bacABCDE-ywfG* [*bac* operon]) (38), and surfactin (39), but only bacillaene (15) and bacilysin antagonized C. jejuni biofilm formation. The PS-216 Δ*bacA* mutant lacking bacilysin but not bacillaene was less antagonistic against C. jejuni than PS-216 WT. Consistently, C. jejuni still formed weak clusters of submerged cells in coculture with Δ*bacA* mutant but not when cocultured with the Δ*pks* mutant that produces bacilysin. This suggests that bacilysin is the most potent B. subtilis antagonist of C. jejuni. Non-ribosomal peptide bacilysin is responsible for growth inhibition of *Xanthomonas* sp. (66), Escherichia coli, and Salmonella enterica and may act by inhibiting cell wall synthesis (64, 67), but it has not been shown before to inhibit C. jejuni. Likewise, surfactin has been put proposed as an antagonist against different Gram-negative and positive pathogens such as Staphylococcus aureus, E. coli, S. enterica, Proteus mirabilis, Shewanella putrefaciens, where the antiadhesive and antibiofilm properties of B. subtilis extracts were identified as lipopeptides, namely, as biosurfactants (e.g., surfactins) (30, 33, 68). However, we show that the Δ*srfAA* mutant still inhibited C. jejuni biofilm formation and/or growth comparable to PS-216 WT, underscoring bacilysin and bacillaene as the main antagonists of C. jejuni.

Bacterial multidrug efflux pumps constitute an important class of resistance determinants against antibiotics (for a review, see references 45 and 56). C. jejuni synthesizes three different efflux pumps—CmeABC, CmeDEF, and CmeGH (48)—which have been mostly investigated from a medical point of view as strategies of resistance to antibiotics that are used in animals and humans (46, 48, 49, 69) but not in a mixed-biofilm setting. Our results show that in coculture with B. subtilis PS-216, the Δ*cmeF* and Δ*cmeR* mutants were more sensitive to inhibition than C. jejuni WT, which was not the case for the Δ*cmeB* and Δ*cmeG* C. jejuni strains. Increased sensitivity of Δ*cmeF* and Δ*cmeR* mutants was confirmed also in coculture with the B. subtilis Δ*pks* mutant (which produces bacilysin but not bacillaene), suggesting that the CmeDEF efflux pump contributes to the C. jejuni defense against bacilysin. Consistently, the Δ*cmeF* mutant showed a 2-fold decrease in resistance to a variety of medically important antibiotics compared to C. jejuni NCTC 11168-WT (53). However, this decrease was not observed if Δ*cmeF* was cocultured with the PS-216 Δ*bacA* mutant, suggesting that the CmeDEF pump does not contribute to defense against bacillaene. Hence, this pump shows specificity. Bacteria often carry several RND efflux pumps; this brings different advantages. Although RND pumps have been recognized for their polyspecificity, they do not provide resistance to the same antibiotics. They may have different substrate specificities (47), which can change depending on the outer membrane’s permeability and the pump’s expression levels (47). This is in line with a dramatic increase of C. jejuni Δ*cmeR* mutant resistance in coculture with the PS-216 Δ*bacA* mutant. This phenotype is also consistent with the previously reported *cmeABC* operon overexpression in the Δ*cmeR* mutant (50, 53), which may also alleviate bacillaene-driven antagonism. However, the Δ*cmeB* mutant with a dysfunctional CmeABC pump showed sensitivity to bacillaene attack similar to that of C. jejuni WT, suggesting that the CmeABC pump at WT levels does not contribute to defense against bacillaene and that it requires a special context to act.

Finally, the third efflux pump, CmeGH, which belongs to the MFS family (54), did not contribute to resistance against B. subtilis antimicrobials in coculture with the PS-216 WT strain. However, in coculture with the Δ*pks* mutant, all four C. jejuni mutants became slightly more sensitive. Although the reason for this effect is unknown and should be addressed in future studies, it is possible that upon deleting one antibiotic (e.g., bacillaene), B. subtilis could increase the production of another (e.g., bacilysin).

Finally, the defects of efflux pump mutants in coculture with B. subtilis were restored in cocultures with the B. subtilis Δ*bacA* Δ*pks* double mutant missing both antibiotics. This result emphasizes the importance of RND family efflux systems in the defense against bacillaene and bacilysin.

In conclusion, B. subtilis PS-216 inhibition of C. jejuni growth and biofilm development depends on polyketide antibiotic bacillaene and dipeptide antibiotic bacilysin. Furthermore, the C. jejuni CmeDEF efflux pump contributes to defense against bacilysin, and the CmeR repressor contributes to the resistance to bacillaene. These findings suggest that multidrug RND pumps of C. jejuni show specificity against antibiotic attack in cocultures. Hence, these results improve our understanding of the mechanisms driving interactions between a potential probiotic B. subtilis PS-216 and an important pathogen, C. jejuni, and will guide future studies *in vivo* in broilers.

## MATERIALS AND METHODS

### Bacterial strains and strain construction.

The strains and genotypes of C. jejuni and B. subtilis strains used in this study and the construction of their mutant derivatives are described and listed in Table 1, including the strains used for the construction of the B. subtilis (15, 70–73) and C. jejuni mutants described previously (74, 75). In multispecies biofilm experiments, C. jejuni NCTC 11168 (WT) and its derivative tagged with a *gfp* gene expressed on the plasmid pWM1007 (76) (WT-GFP), obtained from the Food Safety and Health Research Unit, Agricultural Research Service, U.S. Department of Agriculture (Albany, CA, USA), were used together with a soil isolate B. subtilis PS-216 WT (77) and its derivatives. B. subtilis PS-216 was tagged with a mKate2 fluorescent protein (RFP) linked to a constitutive promoter (P_43_) integrated into the *sacA* locus (utilization of sucrose; *sacA*::P_43_*-mKate2*; Kn) (72) (Table 1). The recombinant strains were constructed by transforming DNA of B. subtilis donor strains or PCR products into B. subtilis recipients using the standard transformation protocol. Transformants were selected on Luria-Bertani (LB) agar supplemented with the following antibiotic concentrations: erythromycin (Erm), 20 μg/mL; kanamycin (Kn), 50 μg/mL; and spectinomycin (Spec), 100 μg/mL. The B. subtilis PS-216 Δ*comA* mutant was constructed by transforming the parent strain with chromosomal DNA isolated from the B. subtilis 168 mutant strain BD1605 (73). The PS-216 Δ*bacA* mutant was constructed by introducing a PCR product via transformation using a B. subtilis 168 Δ*bacA* mutant from the single gene inactivation library and amplified by specific primers (5pL/3pR) (Table 2) (70) as the DNA template. The PS-216 Δ*pks* mutant was constructed by using a PCR fragment amplified from chromosomal DNA isolated from the B. subtilis PSK0178 mutant strain with the deletion of the entire *pks* gene cluster using the PksX1/PksX4 primer pair (Table 2) (71). The PS-216 Δ*srfAA* Δ*pks* and PS-216 Δ*srfAA* Δ*bacA* double mutants were constructed by transforming a purified PCR product from a B. subtilis PSK0178 Δ*pks* mutant strain (71) and a B. subtilis BKE37740 Δ*bacA* mutant strain (70) into the PS-216 Δ*srfAA* strain (15). The PS-216 Δ*pks* Δ*bacA* double mutant was constructed by using purified PCR product from the B. subtilis BKE37740 mutant from a single gene inactivation library (70) in the B. subtilis PS-216 Δ*pks* mutant. B. subtilis mutant strains (Δ*bacA*, Δ*pks*, Δ*srfAA* Δ*pks*, Δ*srfAA* Δ*bacA*, and Δ*pks* Δ*bacA*) were first selected on agar plates supplemented with antibiotics as described above. Next, chromosomal DNA from transformants was isolated and screened by PCR using specific forward and reverse primer pairs (Table 2) to confirm that transformants carried a deletion compared to the PS-216 WT strain. The B. subtilis Δ*comA* mutant strain, along with antibiotic selection on an agar plate, was confirmed by a similar phenotype compared to the parental B. subtilis 168 Δ*comA* strain and a different phenotype compared to the PS-216 WT strain. To construct *sacA*::P*_43_*-*mKate2* reporter fusion strains, we transformed B. subtilis PS-216 Δ*pks*, PS-216 Δ*bacA*, and PS-216 Δ*pks* Δ*bacA* strains with plasmid DNA pMS17, as previously described (72) (Table 1). Strains tagged with mKate2 fluorescent protein linked to a constitutive promoter integrated in *sacA* were, after selection on agar plates, supplemented with antibiotic confirmed for red fluorescence using a fluorescent stereomicroscope (CH9435, type DFC425 C; Leica Microsystems, Wetzlar, Germany) equipped with filter sets ET mCherry MZ10 with excitation filter ET560/40 nm and emission filter ET630/75 nm.

**TABLE 2:** PCR primers and amplification protocols

Primer	B. subtilis targeted gene	Sequence (5′−3′)^*a*^	Annealing temp (°C)	GC content (%)	Source or reference
5pL	*bacA*	F-GGC GAT AAA TAC TCC AGA GAA CTG	58.7	45.8	70
3pR		R-AAA TTG ACT TGC AGC ACC TTG	58.7	42.9	
PksX1	*pks*	F-GAA TAC GTA GCG TAC AGC AAG CC	62	52.2	71
PksX4		R-AAA CGG TTC GGA GCC ACA TAT CC	62	52.2	

a F-, upstream primer; R-, downstream primer.

### Bacterial growth conditions.

C. jejuni NCTC 11168 strain (WT) and its mutants were subcultured from the stock (–80°C). C. jejuni WT was cultivated on Karmali agar (Oxoid, UK) with the selective supplement SR1607E (Oxoid). C. jejuni mutants were cultivated on Müller-Hinton agar (MHA) with appropriate antibiotics supplemented with Kn at 30 μg/mL or Cm at 4 μg/mL, while WT-GFP was constitutively expressed using green fluorescent protein (GFP) on plasmid pWM1007 on MHA medium supplemented with Kn at 50 μg/mL. All C. jejuni cultures were sustained at 42°C under microaerobic conditions using Genbag sachets (bioMérieux). B. subtilis PS-216 and its mutants were subcultured from the stock (–80°C) by cultivation on MHA or MHA medium plus appropriate antibiotics—spectinomycin (Spec), 100 μg/mL; erythromycin (Erm), 20 μg/mL; and kanamycin (Kn), 50 μg/mL—for 24 h. To determine colony counts (CFU/mL) of the B. subtilis strains in mono- or coculture, the samples were subcultured on MHA and MHA medium supplemented with appropriate antibiotics at 28°C for 24 h and under aerobic conditions, which is selective against C. jejuni. The C. jejuni colony counts (CFU/mL) were determined on Karmali agar incubated at 42°C for 24 h under microaerobic conditions.

All B. subtilis*-*C. jejuni coculture (biofilm) experiments were routinely performed in a controlled atmosphere under static microaerobic conditions (Genbag sachets; bioMérieux) at 42°C using standard MHB. Monocultures of both strains were also prepared for control and incubated under the same conditions.

### Multispecies biofilms.

The method to grow cocultures was described previously (15). Briefly, C. jejuni (NCTC 11168 WT or mutants) and B. subtilis (PS-216 WT or PS-216 mutants) were mixed at a ratio of 10:1 in 5 mL of MHB medium, followed by incubation under static microaerobic conditions (Genbag sachets; bioMérieux) at 42°C that support the biofilm development of both species when grown in monocultures. The colony counts were determined at 0 h and after 24 h of coincubation. At 24 h, the biofilms were disrupted by vortexing and strong pipetting before the CFU count was determined on Karmali agar and MHA, as described above.

### Spatial distribution (CLSM) of *B. subtilis* and *C. jejuni* cells in coculture biofilm assay.

Mono- and multispecies biofilms of B. subtilis PS-216 WT and mutant strains labeled with mKate2 and C. jejuni WT-GFP (Table 1) were grown in MHB medium in 96-well microtiter plates (Greiner CELLSTAR) as described previously (15). Strains in coculture were mixed at a ratio of 1:1 (in 100 μL) and were incubated under static, microaerobic conditions at 42°C for 24 h.

Biofilms were investigated as previously described (15) with minor changes and upgrades in the methodology. The spatial distribution and structural properties of B. subtilis and C. jejuni biofilms in mono- and coculture were investigated using CLSM (with the inverted microscope AxioVision Z1, LSM800; Zeiss, Germany) by growing strains (Table 1) as described previously (15). Excitation of GFP was performed at 488 nm with an argon laser, and the emitted fluorescence was recorded at 400 to 580 nm. Excitation of the RFP (mKate2) was performed at 561 nm, and the emitted fluorescence was recorded at 580 to 700 nm. The laser intensities and GaAsP detector gain were 4% and 800 V and 4.5% and 650 V for mKate2 (RFP) and GFP, respectively. The pinhole size was 58 mm. To generate images of the biofilms, 3.5-μm z-stacks (height) were generated for each biological sample. The sizes of the acquired images were typically 1.300 × 1.300 pixels with 16-bit color depth, and microtiter wells were scanned using a 20×/0.4-numerical-aperture (NA) objective. Zen 2.3 software (Carl Zeiss) was used for image acquisition and visualization. The noise on the acquired CLSM images was reduced by applying a single pixel filter (threshold = 1.5). The biofilm thickness in μm was measured directly from ortho view in the Zen 2.3 software (Carl Zeiss).

### Statistical analysis.

To evaluate the influence of cocultivation on the growth of B. subtilis and C. jejuni strains, statistical significance was assessed by a two-sample *t* test (equal variance not assumed [Welch correction]) using raw data or nonparametric/Mann-Whitney test (when the population data did not have a normal distribution). Probability values smaller than 0.05 (*p < *0.05) were considered statistically significant. Three to eight biological and up to three technical replicates were used for all experiments. The data are presented as means ± the standard deviations of the mean. The entire analysis was performed using OriginPro 2020 (OriginLab Corp., Northampton, MA). For the CLSM analysis, we performed five biological experiments with five technical replicates (five wells). CLSM analysis was performed in three different position spots in each well where biofilms were grown; in total, 15 analyses per biological experiment were performed.
