# Supplementary material for: Impact of Bacillus subtilis Antibiotic Bacilysin and Campylobacter jejuni Efflux Pumps on Pathogen Survival in Mixed Biofilms
Source: Microbiol Spectr. 2022 Aug 8;10(4):e02156-22. doi: 10.1128/spectrum.02156-22 (PMC9430781; doi:10.1128/spectrum.02156-22)
Supplement: Supplemental file 1 — Fig. S1 to S3. Download spectrum.02156-22-s0001.pdf, PDF file, 0.9 MB [file spectrum.02156-22-s0001.pdf]

SUPPLEMENTAL MATERIAL

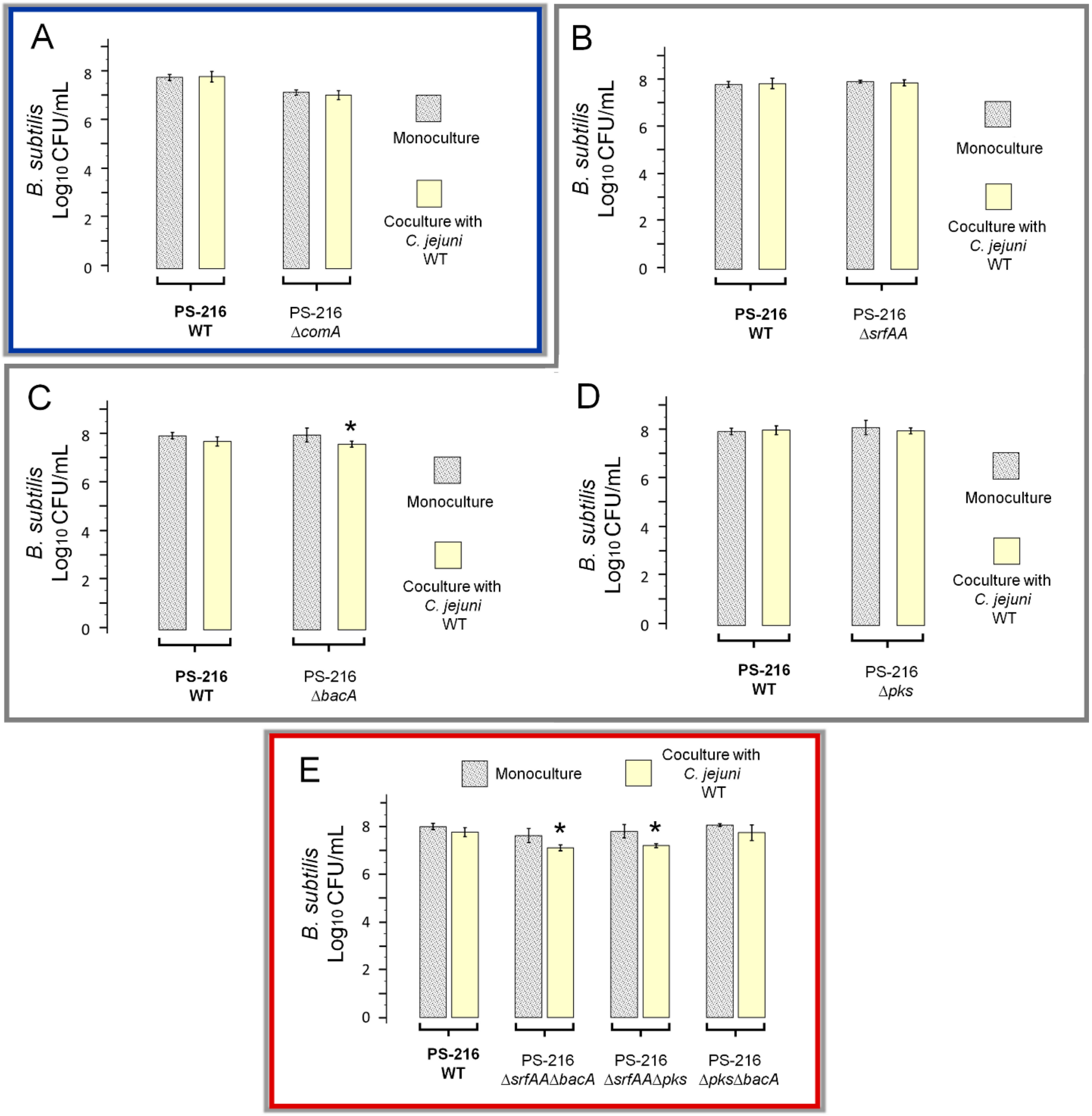

S 1 Inhibition of different *B. subtilis* PS-216 mutants involved in antibiotic synthesis during coculture with *C. jejuni* NCTC 11168 strain. PS-216 mutants in genes involved in transcriptional regulatory protein ComA and in nonribosomal/polyketide synthesis [bacillaene (*pks*), bacilysin (*bacA*), surfactin (*srfAA*)]. (A) *B. subtilis* mutant in gene *comA* encoding transcriptional regulatory

protein ComA during mono- and coculture with *C. jejuni*. (B) *B. subtilis* mutant in gene *srfAA* involved in nonribosomal peptide synthesis of surfactin during mono- and coculture with *C. jejuni*. (C) *B. subtilis* mutant in gene *bacA* involved in nonribosomal peptide synthesis of bacilysin during mono- and coculture with *C. jejuni*. (D) *B. subtilis* mutant in *pks* locus involved in polyketide synthesis of bacillaene during mono- and coculture with *C. jejuni*. (E) *B. subtilis* double mutants in locus involved in polyketide synthesis of bacillaene as well as genes involved in nonribosomal synthesis of surfactin and bacilysin during mono- and coculture with *C. jejuni*. Samples containing biofilm and broth were vortexed prior to plating. Results are presented as colony counts. Three biological and up to three technical repeats were used. The error bars represent the ± standard deviation of the mean value. * represent statistically significant values. Data were statistically evaluated using two-sample t-test, see materials and methods for details.

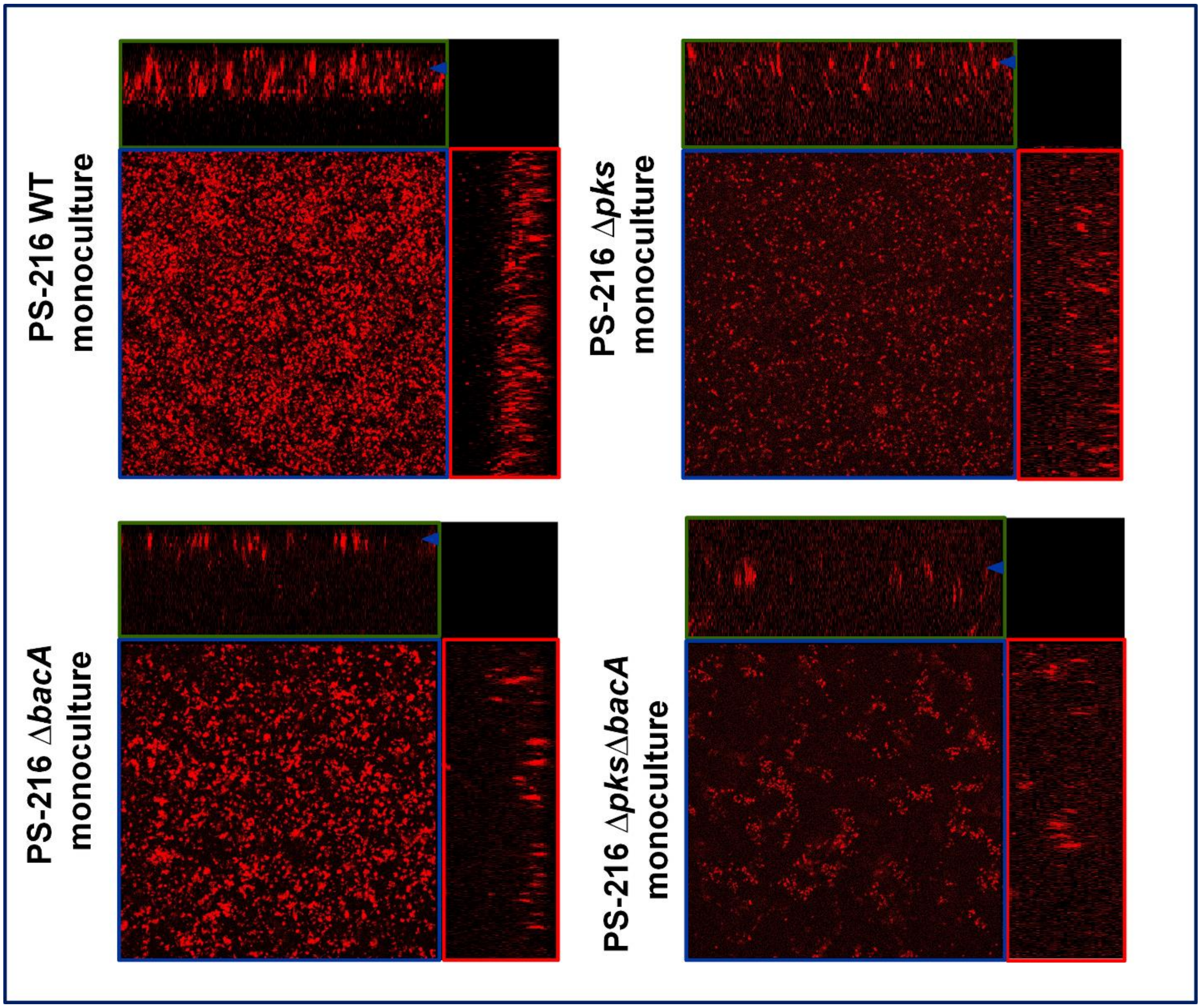

S 2 Orthogonal view of the *B. subtilis* submerged biofilm monocultures of *B. subtilis* PS-216 WT and indicated three PS-216 mutants (Δ*pks*, Δ*bacA*, Δ*pks*Δ*bacA*). Biofilms were grown under static conditions at 42°C in MHB medium as monocultures and analyzed by CLSM. The blue square represents the top-down view of the Z stack confocal image. The spatial resolution involves X and Y dimensions (orthogonal to the projection of axis), which represent the cross-sections of the submerged biofilm and combine 28 Z stack images within 100 μm biofilm depth (orthogonal images/view).

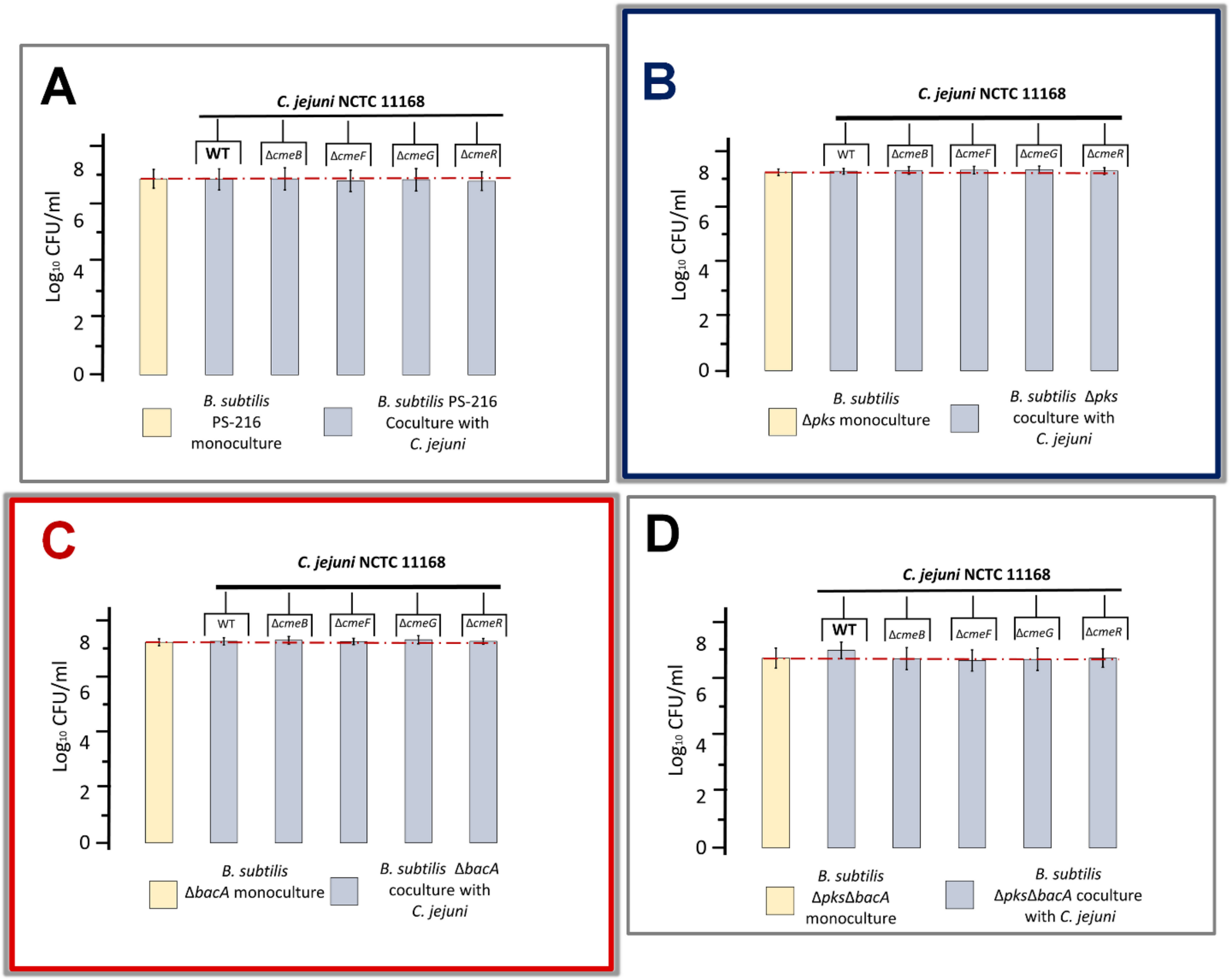

S 3 *B. subtilis* growth was measured as colony counts during static mono- and coculture after 24 h with four different mutants of *C. jejuni*. (A) *B. subtilis* PS-216 WT growth during coculture with four different mutants of *C. jejuni* in loci responsible for proper function of bacterial cell efflux apparatus system is presented. Eight biological and up to three technical repeats for each biological replicate were performed. (B) *B. subtilis* Δ*pks* mutant in locus involved in polyketide synthesis of bacillaene during mono- and coculture with four *C. jejuni* efflux pump mutants. (C) *B. subtilis* Δ*bacA* mutant in locus involved in nonribosomal synthesis of bacilysin during mono- and coculture with four *C. jejuni* efflux pump mutants. Five biological and up to three technical repeats were performed. (D) *B. subtilis* mutant in *pks* locus involved in polyketide synthesis of bacillaene

and in gene *bacA* involved in nonribosomal synthesis of bacilysin (PS-216 Δ*pks*Δ*bacA*) during mono- and coculture with four *C. jejuni* efflux pump mutants. Samples containing biofilm and broth were vortexed prior to plating. Results are presented as colony counts. The error bars represent the ± standard deviation of the mean value. Data were statistically evaluated using two-sample t-test, see materials and methods for details.
